# No association between serum cholesterol and death by suicide in patients with schizophrenia, bipolar affective disorder, or major depressive disorder

**DOI:** 10.1186/1744-9081-9-45

**Published:** 2013-12-05

**Authors:** Subin Park, Ki Kyoung Yi, Riji Na, Ahyoung Lim, Jin Pyo Hong

**Affiliations:** 1 Department of Psychiatry, Seoul National University Hospital, Seoul, Korea; 2 Department of Psychiatry, Asan Medical Center, Ulsan University College of Medicine, 388-1 Pungnap-2dong Songpa-gu, Seoul 138-736, South Korea

**Keywords:** Suicide, Laboratory markers, Schizophrenia, Bipolar affective disorder, Depressive disorder

## Abstract

**Background:**

Previous research on serum total cholesterol and suicidality has yielded conflicting results. Several studies have reported a link between low serum total cholesterol and suicidality, whereas others have failed to replicate these findings, particularly in patients with major affective disorders. These discordant findings may reflect the fact that studies often do not distinguish between patients with bipolar and unipolar depression; moreover, definitions and classification schemes for suicide attempts in the literature vary widely.

**Methods:**

Subjects were patients with one of the three major psychiatric disorders commonly associated with suicide: schizophrenia, bipolar affective disorder, and major depressive disorder (MDD). We compared serum lipid levels in patients who died by suicide (82 schizophrenia, 23 bipolar affective disorder, and 67 MDD) and non-suicide controls (200 schizophrenia, 49 bipolar affective disorder, and 175 MDD).

**Results:**

Serum lipid profiles did not differ between patients who died by suicide and control patients in any diagnostic group.

**Conclusions:**

Our results do not support the use of biological indicators such as serum total cholesterol to predict suicide risk among patients with a major psychiatric disorder.

## Background

Several studies have shown an association between suicidal behavior and low levels of plasma total cholesterol [1-4]. Cholesterol is essential for the cell membrane stability and neurotransmission in central nervous system [5]. Relationship between serum cholesterol and suicidal behavior is explained by the hypothesis that reduced serum cholesterol level may be associated reduced central serotonergic activity by changes in viscosity and functions of serotonin receptors and transporters [6,7].

Recently, Freemantle et al. [8] compared synaptosomal cholesterol and phospholipid levels in suicide completers and controls and found that cholesteryl ester hydrolase was significantly increased in people who committed violent suicides and was associated with alterations in brain phospholipids. However, several studies conducted in patients with a major affective disorder have found no significant association between serum cholesterol and suicide [9-12].

These discordant findings may reflect the fact that studies often do not distinguish between patients with bipolar and unipolar depression [5]. Moreover, the definitions and classification schemes for suicide attempts vary widely (e.g., the distinction between lethality and/or intent to die), which may contribute to inconsistent results across studies [13]. For example, Deisenhammer et al. [14] found no significant difference in lipid levels between patients with and without a history of attempted suicide; however, the authors reported a lower total cholesterol level in patients who committed violent suicides compared with those who poisoned themselves. Furthermore, association between cholesterol level and suicidal behavior was mostly investigated in suicide attempters rather than suicide completers [9], although suicide completers may be a more homogeneous group than suicide attempters [6].

In view of the limited data available in the suicide completers with a specific psychiatric disorder, we compared serum lipid metabolites including serum total cholesterol between patients who died by suicide and non-suicide controls in each group of three major psychiatric disorders commonly associated with suicide: schizophrenia, bipolar affective disorder, and Major depressive disorder (MDD).

## Methods

### Subjects and procedures

Using electronic medical records, we identified all patients who were admitted to a psychiatric ward at a university hospital in Seoul, Korea, for schizophrenia (N = 1562), bipolar affective disorder (N = 728), or MDD (N = 1455) between January, 1989, to December, 2006. All identified patients were older than 18 years of age at the time of admission. The medical record requirements of the participating hospital included one primary diagnosis and several auxiliary diagnoses based on the International Classification of Disease, 10^th^ edition (ICD-10) coding in a discharge summary, which had been recorded by an attending board-certified psychiatrist after close inpatient observation, neuropsychological test, and clinical interview. The diagnosis used for individuals with multiple psychiatric disorders was the primary ICD-10 diagnosis provided in the electronic medical record.

Information about whether or not the patients were alive on December 31, 2009, was obtained from the database of the Korea National Statistical Office. For deceased patients, cause of death was also obtained from the database of the Korea National Statistical Office. Suicide decedents were identified as those with ICD-9 codes E950-E959 between 1989-1998 and those with ICD-10 codes X60-X84 between 1999-2005*.* Of the 1,562 patients with schizophrenia, 84 had died by suicide prior to December 31, 2009. Of the 728 patients with bipolar affective disorder, 30 had died by suicide, and of the 1,455 patients with MDD, 72 had died by suicide prior to December 31, 2009. Among these suicide completers, we excluded patients who lacked fasting laboratory values, had a substance use disorder or an eating disorder, or took cholesterol-lowering drugs. Among 84 patients with schizophrenia, 1 had taking cholesterol lowering agent, 1 had a substance use disorder. Among 30 patients with bipolar disorder, 7 patients had eating disorder. Among 72 patients with major depressive disorder, 2 patients taking cholesterol lowering drug, 3 patients had a substance use disorder. The remaining 82 patients with schizophrenia, 23 patients with bipolar affective disorder, and 67 patients with MDD formed the study sample. As a non-suicide control group, we randomly selected two age (at the time of admission) - and sex-matched controls to each patient who were alive on December 31, 2009. Patients with past history of suicide attempt were excluded from control group, and other exclusion criteria of the control group were same as the suicidal group. Finally, the 164 patients with schizophrenia, 46 patients with bipolar affective disorder, and 134 patients with MDD formed the control group sample.

Three psychiatric residents reviewed the electronic medical records and recorded socio-demographic (age at the time of admission, sex, body mass index, and socio-economic status) and clinical (age at first admission, number of admissions, and history of previous suicide attempts) characteristics. The lipid metabolites (triglyceride, total cholesterol, high-density lipoprotein cholesterol, glucose) at the time of admission were recorded. For patients who had recurrent admission, laboratory values of last admission were used. Atypical antipsychotic drug used at the time of admission was also recorded, because it might be associated with hypertriglyceridemia, hypercholesterolemia, and hyperglycemia [15].

The study was approved by the institutional review board for human subjects at the Asan Medical Center, Ulsan University College of Medicine.

### Statistical analysis

We compared socio-demographic and clinical characteristics and serum lipid metabolites between patients who died by suicide and controls in each diagnostic group using an independent t-test for continuous variables and a chi-square test for categorical variables. All statistical analyses were performed using SPSS (version 21.0; SPSS Inc., Chicago, IL), with statistical significance defined as an alpha level of 0.05

## Results

Table 1 shows demographic and clinical characteristics of participants. Schizophrenia patients who died by suicide were composed of 58 paranoid type (70.7%), 3 disorganized type (3.7%), 1 catatonic type (1.2%), and 17 undifferentiated type (20.7%), and 3 residual type (3.7%) and those who did not were composed of 124 paranoid type (75.6%), 3 disorganized type (1.8%), 5 catatonic type (3.0%), and 27undifferentiated type (16.5%) and 5 residual type (3.0%). There was no significant difference in subtypes of the disorder between schizophrenia patients who died by suicide and controls (p = 0.669). All patients with bipolar disorder suffered from bipolar I disorder. There were no significant differences in socio-demographic and clinical characteristics as well as comorbid medical disease between patients who died by suicide and controls in any diagnostic group. Atypical antipsychotics, known to be cause hypertriglyceridemia, hypercholesterolemia, and hyperglycemia, were used only in small number of patients, because most patients were admitted when their symptoms were developed or aggravated without any medication and laboratory test were conducted before starting medication. There were no significant differences in use of atypical antipsychotics between patients who died by suicide and controls in any diagnostic group.

**Table 1:** Characteristics of schizophrenia, bipolar affective disorder, and major depressive disorder patients who died by suicide and those who did not

	**Schizophrenia**			**Bipolar affective disorder**			**Major depressive disorder**		
	**Suicide**	**Non-suicide**	**p-value**	**Suicide**	**Non-suicide**	**p-value**	**Suicide**	**Non-suicide**	**p-value**
	N = 82	N = 164		N = 23	N = 46		N = 67	N = 134	
Age at laboratory tests (years), mean ± SD	31.1 ± 9.1	31.5 ± 8.6	0.699^a^	36.5 ± 11.6	35.9 ± 10.0	0.830^a^	52.6 ± 15.2	54.1 ± 15.2	0.519^a^
Age at first admission (years)	29.1 ± 9.3	29.3 ± 8.9	0.859^a^	34.0 ± 11.6	32.7 ± 10.4	0.620^a^	51.6 ± 15.1	53.2 ± 14.8	0.494^a^
Number of admissions, mean ± SD	2.2 ± 2.3	2.4 ± 2.5	0.638^a^	2.5 ± 1.8	2.5 ± 2.1	>0.99^a^	1.6 ± 1.3	1.6 ± 1.0	0.895^a^
Body mass index (kg/m^2^), mean ± SD	22.1 ± 3.7	23.1 ± 4.1	0.086^a^	23.8 ± 3.6	23.9 ± 3.0	0.93^a^	22.8 ± 3.2	22.9 ± 3.5	0.894^a^
Sex, male, n (%)	42(51)	80(49)	0.787^b^	14(61)	29(63)	>0.99^b^	30(45)	56(42)	0.763^b^
Socio-economic status, n (%)			0.479^b^			0.457^b^			0.259^b^
High	19(27)	33(21)		3(14)	12(27)		13(24)	39(31)	
Middle	32(45)	84(53)		12(55)	22(50)		28(51)	66(53)	
Low	20(28)	41(26)		7(32)	10(23)		14(25)	20(16)	
Use of atypical antipsychotics, n (%)	16 (19.5)	43 (26.2)	0.245^b^	4 (17.4)	11 (23.9)	0.536^b^	4 (6.0)	7 (5.2)	0.826^b^
Comorbid medical disease									
None	75(91.5)	155(94.5)	0.361^b^	17(73.9)	40(87.0)	0.178^b^	47(70.1)	81(60.4)	0.178^b^
Endocrine disease	2(2.4)	1 (0.6)		0	0		4(6.0)	13(9.7)	
Cardiovascular disease	0	1(0.6)		2(8.7)	2(3.3)		12(17.9)	22(16.4)	
Liver disease	0	3(1.8)		1(4.3)	0		0	3(2.2)	
Neurological disease	3(3.7)	1(0.6)		2(8.7)	1(2.2)		3(4.5)	3(2.2)	
Cancer	0	0		0	0		0	3(2.2)	
Others^c^	2(2.4)	3(1.8)		1(4.3)	3(6.5)		1(1.5)	9(6.7)	

^a^, t-test; ^b^, X^2^ test; ^c^, Others include cystitis, fracture, rheumatoid arthritis, blind, pneumothorax, and pulmonary tuberculosis.

Table 2 shows serum lipid metabolites of patients who died by suicide and controls in each diagnostic group. Lipid profiles did not differ between patients who died by suicide and controls in any diagnostic group.

**Table 2:** Lipid levels of schizophrenia, bipolar affective disorder and major depressive disorder patients who died by suicide and those who did not

	**Schizophrenia**			**Bipolar affective disorder**			**Major depressive disorder**		
	**Suicide**	**Non-suicide**	**p-value**	**Suicide**	**Non-suicide**	**p-value**	**Suicide**	**Non-suicide**	**p-value**
	N = 82	N = 164		N = 23	N = 46		N = 67	N = 134	
Total cholesterol(mg/dL)	162.9 ±36.1	172.3 ± 41.1	0.082	165.2 ± 30.8	167.9 ± 42.9	0.791	180.9 ± 40.0	180.6 ± 35.4	0.945
Glucose (mg/dL)	94.1 ± 29.5	96.1 ± 23.9	0.567	93. 9 ± 20.3	103.9 ± 27.8	0.135	108.5 ± 44.2	101.0 ± 24.5	0.202
Triglyceride (mg/dL)	104.9 ± 54.9	129.5 ± 74.2	0.124	105.9 ± 88.6	128.1 ± 95.2	0.467	107.8 ± 57.9	131.0 ± 74.1	0.124
HDL cholesterol(mg/dL)	47.2 ± 17.1	48.3 ± 13.3	0.745	61.7 ± 19.4	53.5 ± 12.5	0.165	49.5 ± 15.5	50.4 ± 15.9	0.795

Data are presented as mean ± standard deviation. P-values were determined by t-test. HDL, high density lipoprotein.

When we compared serum lipid metabolites across diagnosis group, patients with MDD showed significantly higher total cholesterol levels (mean = 180.7, SD = 36.9) compared to patients with schizophrenia (mean = 169,2, SD = 39.7) or bipolar affective disorders (mean = 167.0, SD = 39.1) (F = 6.04, p = 0.003). However, after adjusting for age, any lipid profiles did not differ between three diagnosis group (adjusted mean ± SE = 173.2 ± 3.2for MDD, 174.5 ± 2,8 for schizophrenia, and169.6 ± 4.6 for bipolar affective disorder, respectively; p = 0.644).

## Discussion

We found no association between death by suicide and the levels of serum lipid metabolites in our sample of inpatients with schizophrenia, bipolar affective disorder, and major depressive disorder (MDD). Thus, our results do not support the use of biological indicators, such as serum total cholesterol level, as predictors of suicide risk in inpatients with a major psychiatric disorder.

Our findings are consistent with those of previous studies that found no association between low serum cholesterol levels and suicide attempts in patients with major affective disorders. For example, Fiedorowicz et al. [9] reported that low serum cholesterol levels did not predict subsequent suicide attempts in a prospective sample of inpatients with unipolar major depression, bipolar depression, or schizoaffective depression. Pompili et al. [12] found no significant difference in serum cholesterol and triglyceride levels between patients with major affective disorders who had been admitted for a medically serious suicide attempt and patients who had not made a recent suicide attempt. In contrast, Papadopoulou et al. [16] reported that serum cholesterol levels were lower in suicide attempters with a major affective disorder than in healthy controls, and that serum cholesterol levels were negatively correlated with suicide intent scores. The authors concluded that serum cholesterol was a biological risk factor for suicidal behavior in affective patients. However, Papadopoulou and colleagues investigated suicide attempts rather than suicide completion and did not distinguish between patients with bipolar and unipolar depression.

In a meta-analysis of 11 large follow-up studies that investigated the link between low serum cholesterol, Lester et al. [4] found that lower cholesterol levels were associated with a small, but statistically significant, increase in the risk of completed suicide. Of those 11 studies, only two were conducted in individuals with mood disorders, and no significant differences in serum cholesterol or serum triglyceride were found between patients with depression who committed suicide and those who were non-suicidal [10,11]. Tsai et al. [17] also reported that there were no significant differences in fasting levels of serum cholesterol or blood sugar between the suicide completers and the living controls among inpatients with bipolar disorder in Taiwan. Our results were consistent with these findings.

It is notable that patients with MDD showed significantly higher total cholesterol levels compared to patients with schizophrenia, bipolar affective disorders. However, after adjusting for age, these significant differences did not remain. Therefore, higher cholesterol level in patients with MDD might be due to their older age than other diagnostic groups, because serum cholesterol level generally increased, about to 60 years old [18].

The present study is limited by its small sample size and lack of laboratory data during follow-up after discharge from hospital. The small number of cases may have reduced the likelihood of finding statistical significance. When we conducted a power analysis (independent t-test, 1-tailed, α=0.05, β=0.80) based on previous studies’ total cholesterol levels of suicidal patients and psychiatric controls [17,19-21], the required sample size to detect a significant group-differences ranged between 14 and 80 per group. Therefore, at least for schizophrenia group, with the given sample size (82 suicides and 164 non-suicides), it is possible to detect clinical relevant differences and the negative finding may not come from a small sample size. Additionally, since the study was conducted in a single university hospital, the findings may not be representative of all Korean patients with major psychiatric disorders. In a university hospital setting, patients with severe disease and with high suicide risk could be over-represented and further community-based study is therefore required. Moreover, as with most retrospective studies, there were no standardized procedures for arriving at diagnoses. In addition, because data were gathered from electronic medical charts, without data from face-to-face interviews, some information on risk factors previously identified, such as symptomatic characteristics (i.e., self-devaluation, hopelessness, and insomnia) and poor adherence to medications [22] have not been available. Finally, non-significant differences in potential confounders between the suicide and non-suicide groups (e.g., previous history of suicide attempt) could have biased our results.

In conclusion, our results do not support the use of biological indicators such as serum total cholesterol to predict suicide risk among patients with a major psychiatric disorder. To clarify these issues, further longitudinal studies on a large sample of patients are need.

## Consent

Informed consent was not needed. Because data does not contain any identifying information.

## Competing interests

The authors declare that they have no competing interests.

## Authors' contribution

JPH designed the study. KKY, RN, AL participated in data collection. SP and KKY analyzed the data and prepared the first draft of the report. JPH supervised the statistical analysis and interpreted the results. SP wrote the final report with input from all the authors. All authors read and approved the final manuscript.
